# *N*-(3-Methyl­phen­yl)succinimide

**DOI:** 10.1107/S1600536810015904

**Published:** 2010-05-08

**Authors:** B. S. Saraswathi, B. Thimme Gowda, Sabine Foro, Hartmut Fuess

**Affiliations:** a Department of Chemistry, Mangalore University, Mangalagangotri 574 199, Mangalore, India; b Institute of Materials Science, Darmstadt University of Technology, Petersenstrasse 23, D-64287 Darmstadt, Germany

## Abstract

In the title compound, C_11_H_11_NO_2_, the dihedral angle between the ring planes is 52.5 (1)°.

## Related literature

For related structures, see: Saraswathi *et al.* (2010**a* ▶,b* ▶).
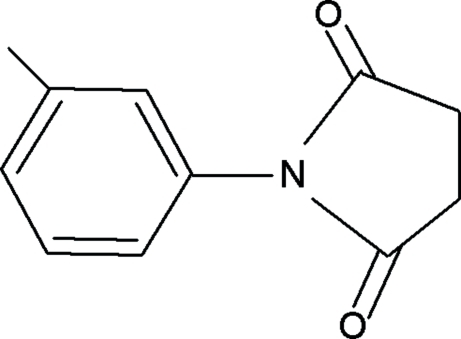


## Experimental

### 

#### Crystal data

C_11_H_11_NO_2_
*M* *_r_* = 189.21
Monoclinic, 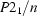

*a* = 7.7906 (9) Å
*b* = 6.6015 (8) Å
*c* = 19.511 (2) Å
β = 100.06 (1)°
*V* = 988.02 (19) Å^3^
*Z* = 4
Mo *K*α radiation
μ = 0.09 mm^−1^
*T* = 299 K
0.32 × 0.16 × 0.14 mm

#### Data collection

Oxford Diffraction Xcalibur diffractometer with a Sapphire CCD detector
Absorption correction: multi-scan (*CrysAlis RED*; Oxford Diffraction, 2009 ▶) *T*_min_ = 0.972, *T*_max_ = 0.988
3757 measured reflections
2000 independent reflections
1453 reflections with *I* > 2σ(*I*)
*R*_int_ = 0.020

#### Refinement

*R*[*F* ^2^ > 2σ(*F* ^2^)] = 0.050
*wR*(*F* ^2^) = 0.168
*S* = 1.18
2000 reflections
128 parameters
H-atom parameters constrained
Δρ_max_ = 0.17 e Å^−3^
Δρ_min_ = −0.18 e Å^−3^

### 

Data collection: *CrysAlis CCD* (Oxford Diffraction, 2009 ▶); cell refinement: *CrysAlis RED* (Oxford Diffraction, 2009 ▶); data reduction: *CrysAlis RED*; program(s) used to solve structure: *SHELXS97* (Sheldrick, 2008 ▶); program(s) used to refine structure: *SHELXL97* (Sheldrick, 2008 ▶); molecular graphics: *PLATON* (Spek, 2009 ▶); software used to prepare material for publication: *SHELXL97*.

## Supplementary Material

Crystal structure: contains datablocks I, global. DOI: 10.1107/S1600536810015904/ng2766sup1.cif

Structure factors: contains datablocks I. DOI: 10.1107/S1600536810015904/ng2766Isup2.hkl

Additional supplementary materials:  crystallographic information; 3D view; checkCIF report

## Figures and Tables

**Fig. 1.**
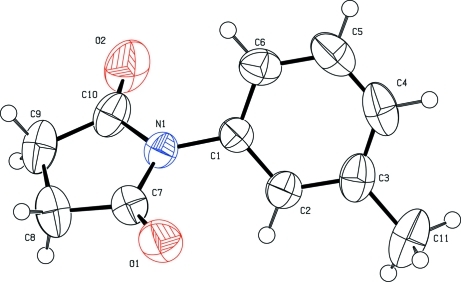
Molecular structure of the title compound, showing the atom labelling scheme. The displacement ellipsoids are drawn at the 50% probability level. The H atoms are represented as small spheres of arbitrary radii.

**Fig. 2.**
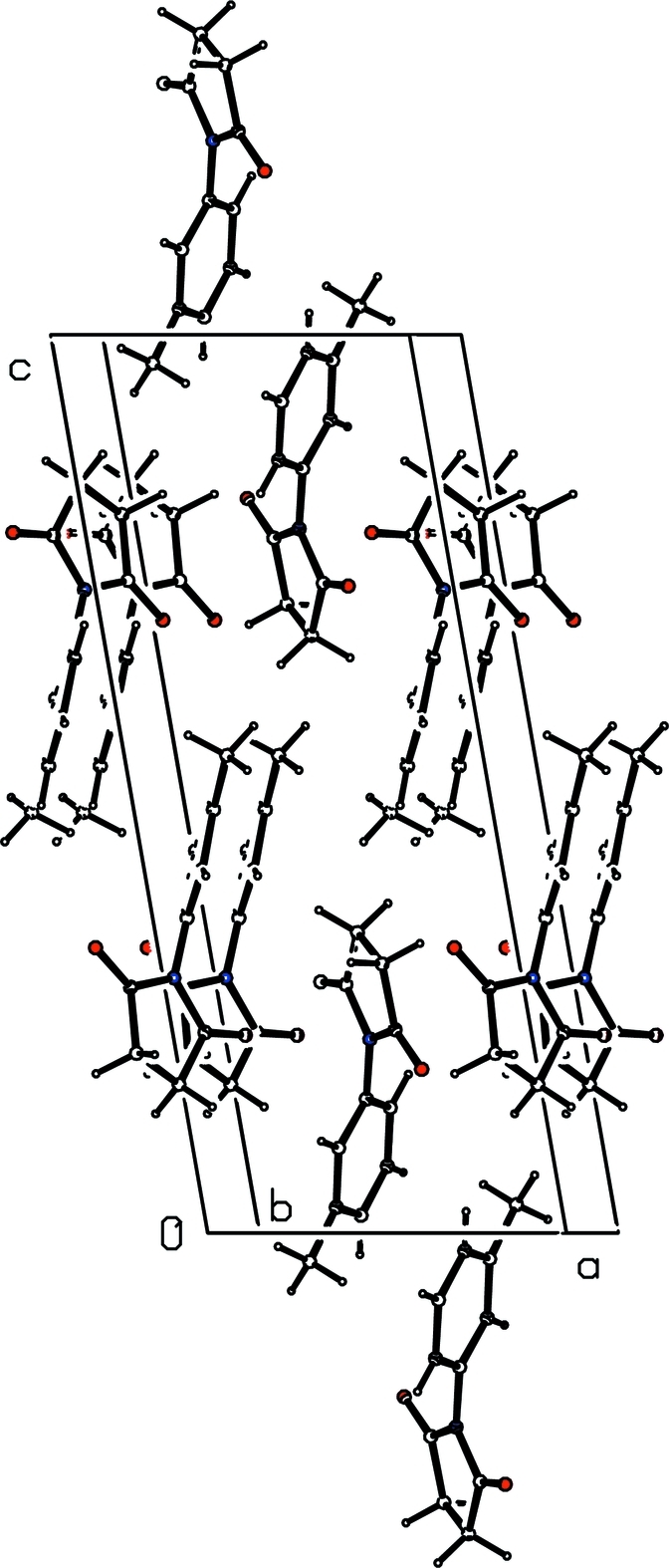
Molecular packing of the title compound.

## supplementary crystallographic information

### Comment

As a part of studying the effect of ring and side chain substitutions on the structures of biologically significant compounds (Saraswathi *et al.*, 2010*a,b*), the crystal structure of *N*,*N*-(3-methylphenyl)succinimide has been determined (Fig.1). In the structure, the molecule is non-planar with the benzene and pyrrolidine rings tilted by 52.5 (1)° with respect to one another, compared to the values of 57.3 (1)° in *N*,*N*-(4-methylphenyl)succinimide (Saraswathi *et al.*, 2010*a*) and 67.7 (1)° jn *N*,*N*- (2,3-dimethylphenyl)succinimide (Saraswathi *et al.*, 2010*b*).

The torsional angles of the groups, C2 - C1 - N1 - C7, C6 - C1 - N1 - C7, C2 - C1 - N1 - C10 and C6 - C1 - N1 - C10 in the molecule are 52.6 (2), -127.0 (2), -123.4 (2) and 57.0 (2)°, respectively, while the torsional angles of the groups, O1 - C7 - N1 - C1, C8 - C7 - N1 - C1, O2 - C10 - N1 - C1 and C9 - C10 - N1 - C1 are 7.7 (3), -171.5 (2), -2.1 (3) and -178.6 (2)°, respectively.

The packing of molecules into layered row like chains along *b*-axis is shown in Fig.2.

### Experimental

The solution of succinic anhydride (0.02 mole) in toluene (25 ml) was treated dropwise with the solution of 3-methylaniline (0.02 mole) also in toluene (20 ml) with constant stirring. The resulting mixture was stirred for one hour and set aside for an additional hour at room temperature for the completion of reaction. The mixture was then treated with dilute hydrochloric acid to remove the unreacted 3-methylaniline. The resultant solid *N*-(3-methylphenyl)succinamic acid was filtered under suction and washed thoroughly with water to remove the unreacted succinic anhydride and succinic acid. It was recrystallized to constant melting point from ethanol.

*N*-(3-methylphenyl)succinamic acid was heated for 2 h and then allowed to cool slowly to room temperature to get the compound, *N*-(3-methylphenyl)succinimide. The purity of the compound was checked and characterized by its infrared spectra.

Rod like colourless single crystals of the compound used in X-ray diffraction studies were grown in ethanolic solution by a slow evaporation at room temperature.

### Refinement

The H atoms were positioned with idealized geometry using a riding model with C—H = 0.93–0.97 Å. Isotropic displacement parameters for the H atoms were set equal to 1.2 U_eq_ (parent atom).

### Crystal data

**Table:**

C_11_H_11_NO_2_	*F*(000) = 400
*M**_r_* = 189.21	*D*_x_ = 1.272 Mg m^−^^3^
Monoclinic, *P*2_1_/*n*	Mo *K*α radiation, λ = 0.71073 Å
Hall symbol: -P 2yn	Cell parameters from 1573 reflections
*a* = 7.7906 (9) Å	θ = 2.6–27.7°
*b* = 6.6015 (8) Å	µ = 0.09 mm^−^^1^
*c* = 19.511 (2) Å	*T* = 299 K
β = 100.06 (1)°	Rod, colourless
*V* = 988.02 (19) Å^3^	0.32 × 0.16 × 0.14 mm
*Z* = 4	

### Data collection

**Table:**

Oxford Diffraction Xcalibur diffractometer with a Sapphire CCD detector	2000 independent reflections
Radiation source: fine-focus sealed tube	1453 reflections with *I* > 2σ(*I*)
graphite	*R*_int_ = 0.020
Rotation method data acquisition using ω and φ scans	θ_max_ = 26.4°, θ_min_ = 2.7°
Absorption correction: multi-scan (*CrysAlis RED*; Oxford Diffraction, 2009)	*h* = −9→9
*T*_min_ = 0.972, *T*_max_ = 0.988	*k* = −8→6
3757 measured reflections	*l* = −24→22

### Refinement

**Table:**

Refinement on *F*^2^	Primary atom site location: structure-invariant direct methods
Least-squares matrix: full	Secondary atom site location: difference Fourier map
*R*[*F*^2^ > 2σ(*F*^2^)] = 0.050	Hydrogen site location: inferred from neighbouring sites
*wR*(*F*^2^) = 0.168	H-atom parameters constrained
*S* = 1.18	*w* = 1/[σ^2^(*F*_o_^2^) + (0.1*P*)^2^] where *P* = (*F*_o_^2^ + 2*F*_c_^2^)/3
2000 reflections	(Δ/σ)_max_ < 0.001
128 parameters	Δρ_max_ = 0.17 e Å^−^^3^
0 restraints	Δρ_min_ = −0.18 e Å^−^^3^

### Special details

**Table:**

Experimental. CrysAlis RED (Oxford Diffraction, 2009) Empirical absorption correction using spherical harmonics, implemented in SCALE3 ABSPACK scaling algorithm.
Geometry. All esds (except the esd in the dihedral angle between two l.s. planes) are estimated using the full covariance matrix. The cell esds are taken into account individually in the estimation of esds in distances, angles and torsion angles; correlations between esds in cell parameters are only used when they are defined by crystal symmetry. An approximate (isotropic) treatment of cell esds is used for estimating esds involving l.s. planes.
Refinement. Refinement of *F*^2^ against ALL reflections. The weighted *R*-factor wR and goodness of fit *S* are based on *F*^2^, conventional *R*-factors *R* are based on *F*, with *F* set to zero for negative *F*^2^. The threshold expression of *F*^2^ > σ(*F*^2^) is used only for calculating *R*-factors(gt) etc. and is not relevant to the choice of reflections for refinement. *R*-factors based on *F*^2^ are statistically about twice as large as those based on *F*, and *R*- factors based on ALL data will be even larger.

### Fractional atomic coordinates and isotropic or equivalent isotropic displacement parameters (Å^2^)

**Table:**

	*x*	*y*	*z*	*U*_iso_*/*U*_eq_	
C1	0.0804 (2)	0.1090 (2)	0.35080 (8)	0.0418 (4)	
C2	0.1645 (2)	−0.0080 (3)	0.40538 (9)	0.0477 (5)	
H2	0.1895	−0.1430	0.3977	0.057*	
C3	0.2117 (2)	0.0744 (3)	0.47137 (10)	0.0590 (5)	
C4	0.1759 (3)	0.2780 (4)	0.48018 (12)	0.0717 (7)	
H4	0.2069	0.3364	0.5240	0.086*	
C5	0.0957 (3)	0.3947 (3)	0.42550 (12)	0.0699 (6)	
H5	0.0744	0.5311	0.4325	0.084*	
C6	0.0468 (3)	0.3106 (3)	0.36036 (10)	0.0552 (5)	
H6	−0.0082	0.3892	0.3234	0.066*	
C7	−0.0722 (2)	−0.1600 (3)	0.27360 (9)	0.0505 (5)	
C8	−0.0778 (3)	−0.2276 (4)	0.20019 (10)	0.0679 (6)	
H8A	−0.1969	−0.2528	0.1775	0.081*	
H8B	−0.0103	−0.3505	0.1987	0.081*	
C9	0.0004 (3)	−0.0551 (4)	0.16546 (10)	0.0710 (7)	
H9A	0.0894	−0.1044	0.1405	0.085*	
H9B	−0.0886	0.0143	0.1329	0.085*	
C10	0.0783 (2)	0.0839 (3)	0.22331 (9)	0.0570 (5)	
C11	0.2939 (3)	−0.0550 (5)	0.53171 (12)	0.0857 (8)	
H11A	0.3588	−0.1625	0.5151	0.103*	
H11B	0.2045	−0.1114	0.5541	0.103*	
H11C	0.3709	0.0264	0.5644	0.103*	
N1	0.02773 (18)	0.0164 (2)	0.28405 (7)	0.0438 (4)	
O1	−0.1388 (2)	−0.2413 (2)	0.31764 (8)	0.0682 (5)	
O2	0.1681 (2)	0.2311 (3)	0.22030 (8)	0.0867 (6)	

### Atomic displacement parameters (Å^2^)

**Table:**

	*U*^11^	*U*^22^	*U*^33^	*U*^12^	*U*^13^	*U*^23^
C1	0.0422 (8)	0.0435 (9)	0.0422 (9)	−0.0036 (7)	0.0142 (7)	−0.0011 (7)
C2	0.0468 (9)	0.0519 (10)	0.0461 (10)	−0.0010 (8)	0.0131 (7)	0.0013 (8)
C3	0.0479 (10)	0.0869 (15)	0.0437 (10)	−0.0122 (10)	0.0123 (8)	−0.0003 (10)
C4	0.0711 (13)	0.0912 (17)	0.0566 (12)	−0.0205 (13)	0.0219 (10)	−0.0293 (12)
C5	0.0807 (14)	0.0573 (12)	0.0786 (16)	−0.0142 (12)	0.0330 (12)	−0.0206 (11)
C6	0.0573 (10)	0.0491 (11)	0.0631 (12)	−0.0030 (9)	0.0218 (9)	0.0037 (9)
C7	0.0442 (9)	0.0552 (11)	0.0521 (11)	−0.0006 (8)	0.0086 (8)	−0.0047 (8)
C8	0.0556 (11)	0.0902 (16)	0.0564 (12)	−0.0069 (11)	0.0057 (9)	−0.0211 (11)
C9	0.0618 (12)	0.1086 (18)	0.0423 (11)	0.0053 (12)	0.0086 (9)	−0.0046 (11)
C10	0.0505 (10)	0.0788 (14)	0.0436 (10)	−0.0027 (10)	0.0134 (8)	0.0103 (9)
C11	0.0681 (14)	0.138 (2)	0.0493 (12)	−0.0046 (15)	0.0058 (10)	0.0168 (13)
N1	0.0427 (7)	0.0491 (8)	0.0400 (8)	−0.0009 (6)	0.0085 (6)	0.0025 (6)
O1	0.0757 (9)	0.0631 (9)	0.0698 (9)	−0.0190 (7)	0.0235 (7)	−0.0006 (7)
O2	0.0944 (12)	0.1052 (13)	0.0646 (10)	−0.0348 (10)	0.0254 (9)	0.0154 (9)

### Geometric parameters (Å, °)

**Table:**

C1—C6	1.375 (3)	C7—N1	1.396 (2)
C1—C2	1.385 (2)	C7—C8	1.494 (2)
C1—N1	1.432 (2)	C8—C9	1.507 (3)
C2—C3	1.387 (3)	C8—H8A	0.9700
C2—H2	0.9300	C8—H8B	0.9700
C3—C4	1.389 (3)	C9—C10	1.498 (3)
C3—C11	1.504 (3)	C9—H9A	0.9700
C4—C5	1.375 (3)	C9—H9B	0.9700
C4—H4	0.9300	C10—O2	1.204 (2)
C5—C6	1.378 (3)	C10—N1	1.386 (2)
C5—H5	0.9300	C11—H11A	0.9600
C6—H6	0.9300	C11—H11B	0.9600
C7—O1	1.205 (2)	C11—H11C	0.9600
			
C6—C1—C2	120.76 (16)	C9—C8—H8A	110.7
C6—C1—N1	120.37 (15)	C7—C8—H8B	110.7
C2—C1—N1	118.87 (15)	C9—C8—H8B	110.7
C1—C2—C3	120.54 (18)	H8A—C8—H8B	108.8
C1—C2—H2	119.7	C10—C9—C8	105.46 (16)
C3—C2—H2	119.7	C10—C9—H9A	110.6
C2—C3—C4	117.90 (19)	C8—C9—H9A	110.6
C2—C3—C11	120.8 (2)	C10—C9—H9B	110.6
C4—C3—C11	121.3 (2)	C8—C9—H9B	110.6
C5—C4—C3	121.34 (19)	H9A—C9—H9B	108.8
C5—C4—H4	119.3	O2—C10—N1	123.72 (18)
C3—C4—H4	119.3	O2—C10—C9	128.29 (17)
C4—C5—C6	120.3 (2)	N1—C10—C9	107.98 (17)
C4—C5—H5	119.8	C3—C11—H11A	109.5
C6—C5—H5	119.8	C3—C11—H11B	109.5
C1—C6—C5	119.10 (19)	H11A—C11—H11B	109.5
C1—C6—H6	120.5	C3—C11—H11C	109.5
C5—C6—H6	120.5	H11A—C11—H11C	109.5
O1—C7—N1	124.49 (16)	H11B—C11—H11C	109.5
O1—C7—C8	127.37 (18)	C10—N1—C7	112.22 (15)
N1—C7—C8	108.13 (16)	C10—N1—C1	124.11 (15)
C7—C8—C9	105.02 (17)	C7—N1—C1	123.56 (13)
C7—C8—H8A	110.7		
			
C6—C1—C2—C3	2.0 (3)	C8—C9—C10—N1	−8.2 (2)
N1—C1—C2—C3	−177.61 (15)	O2—C10—N1—C7	−178.5 (2)
C1—C2—C3—C4	−1.6 (3)	C9—C10—N1—C7	2.2 (2)
C1—C2—C3—C11	176.53 (16)	O2—C10—N1—C1	−2.1 (3)
C2—C3—C4—C5	0.2 (3)	C9—C10—N1—C1	178.57 (15)
C11—C3—C4—C5	−177.9 (2)	O1—C7—N1—C10	−175.92 (19)
C3—C4—C5—C6	0.8 (3)	C8—C7—N1—C10	5.0 (2)
C2—C1—C6—C5	−1.0 (3)	O1—C7—N1—C1	7.7 (3)
N1—C1—C6—C5	178.66 (16)	C8—C7—N1—C1	−171.46 (15)
C4—C5—C6—C1	−0.4 (3)	C6—C1—N1—C10	57.0 (2)
O1—C7—C8—C9	171.10 (19)	C2—C1—N1—C10	−123.42 (19)
N1—C7—C8—C9	−9.8 (2)	C6—C1—N1—C7	−127.04 (18)
C7—C8—C9—C10	10.8 (2)	C2—C1—N1—C7	52.6 (2)
C8—C9—C10—O2	172.5 (2)		
